# Ethaninidothioic acid (R5421) is not a selective inhibitor of platelet phospholipid scramblase activity

**DOI:** 10.1111/bph.15152

**Published:** 2020-06-30

**Authors:** Sarah Louise Millington‐Burgess, Arkadiusz M. Bonna, Taufiq Rahman, Matthew Thomas Harper

**Affiliations:** ^1^ Department of Pharmacology University of Cambridge Cambridge UK; ^2^ CambCol Laboratories Ely Cambridgeshire UK

## Abstract

**Background and Purpose:**

Ethaninidothioic acid (R5421) has been used as a scramblase inhibitor to determine the role of phospholipid scrambling across a range of systems including platelet procoagulant activity. The selectivity of R5421 has not been thoroughly studied. Here, we characterised the effects of R5421 on platelet function and its suitability for use as a scramblase inhibitor.

**Experimental Approach:**

Human platelet activation was measured following pretreatment with R5421 and stimulation with a range of agonists. Phosphatidylserine exposure was measured using annexin V binding. Integrin α_IIb_β_3_ activation and α‐granule release were measured by flow cytometry. Cytosolic Ca^2+^ signals were measured using Cal520 fluorescence. An in silico ligand‐based screen identified 16 compounds which were tested in these assays.

**Key Results:**

R5421 inhibited A23187‐induced phosphatidylserine exposure in a time‐ and temperature‐dependent manner. R5421 inhibited Ca^2+^ signalling from the PAR1, PAR4 and glycoprotein VI receptors as well as platelet α_IIb_β_3_ integrin activation and α‐granule release. R5421 is therefore not a selective inhibitor of platelet scramblase activity. An in silico screen identified the pesticide thiodicarb as similar to R5421. It also inhibited platelet phosphatidylserine exposure, Ca^2+^ signalling from the PAR1 and glycoprotein VI, α_IIb_β_3_ activation and α‐granule release. Thiodicarb additionally disrupted Ca^2+^ homeostasis in unstimulated platelets.

**Conclusion and Implications:**

R5421 is not a selective inhibitor of platelet scramblase activity. We have identified the pesticide thiodicarb, which had similar effects on platelet function to R5421 as well as additional disruption of Ca^2+^ signalling which may underlie some of thiodicarb's toxicity.

## Graphical abstract

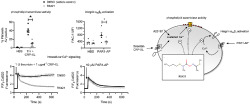

**Abbreviations**
AADACL1: arylacetamide deacetylase‐like 1
ACD: acid citrate dextrose
CRP‐XL: cross‐linked collagen‐related peptide
FITC: fluorescein isothiocyanate
GP: glycoprotein
HBS: HEPES‐buffered saline
PAR‐1AP: protease‐activated receptor 1‐activating peptide
PAR‐4AP: protease‐activated receptor 4‐activating peptide
PE: phycoerythrin
PFA: paraformaldehyde
PIP_3_: phosphoinositide 3,4,5 trisphosphate
PS: phosphatidylserine
R5421: ethaninidothioic acid

**What is already known**

- Platelet phospholipid scramblase activity is an important player in thrombosis.
- Ethaninidothioic acid (R5421) is believed to be a selective inhibitor of platelet phospholipid scramblase activity.

**Wha this study adds**

- We show that R5421 is not a selective inhibitor of scramblase activity in platelets.
- Thiodicarb, a pesticide with similar structure to R5421, also has multiple effects on platelets.

**What is the clinical significance**

- A platelet phospholipid scramblase inhibitor may be a useful anti‐thrombotic.
- R5421 is not a suitable scaffold to develop such an inhibitor.

## 1 INTRODUCTION

Platelets play a central role in arterial thrombosis, the main proximal cause of acute coronary syndrome (Libby, 2013). Inhibition of platelet activity is therefore a key antithrombotic strategy in cardiovascular disease patients (McFadyen, Schaff, & Peter, 2018). Platelets are activated at sites of atherosclerotic plaque rupture, leading to extensive platelet aggregation mediated by integrin αIIbβ_3_ (glycoprotein [GP] IIb/IIIa) (Huang et al., 2019). In addition, a subpopulation of activated platelets shows procoagulant activity, amplifying and propagating local activation of the coagulation cascade (van der Meijden & Heemskerk, 2019).

Clinically used anti‐platelet drugs primarily act by inhibiting platelet aggregation (Jamasbi et al., 2017). These drugs include the COX inhibitor aspirin, the P2Y_12_ receptor antagonists clopidogrel, prasugrel, ticagrelor and cangrelor, the PDE inhibitor dipyridamole and the integrin αIIbβ_3_ inhibitors, eptifibatide, tirofiban and abciximab (McFadyen et al., 2018). All of these are associated with increased bleeding risk (Swieringa, Kuijpers, Heemskerk, & van der Meijden, 2014).

An alternative antithrombotic approach could be to inhibit platelet procoagulant activity. Procoagulant platelets expose phosphatidylserine (PS) on their surface, forming a binding site for the tenase and prothrombinase coagulation complexes (De Witt, Verdoold, Cosemans, & Heemskerk, 2014). These procoagulant platelets are a distinct subset of the activated platelet population, separate from pro‐aggregatory platelets with active integrin αIIbβ_3_ (van der Meijden & Heemskerk, 2019). Phosphatidylserine exposure in procoagulant platelets is driven by sustained intracellular Ca^2+^ signalling, opening of the mitochondrial permeability transition pore and collapse of mitochondrial membrane potential (Choo, Saafir, Mkumba, Wagner, & Jobe, 2012; Jobe et al., 2008). This leads to activation of a Ca^2+^‐dependent scramblase, transmembrane protein 16F (TMEM16F), collapse of plasma membrane phospholipid asymmetry and net movement of phosphatidylserine to the outer leaflet of the platelet membrane (Fujii, Sakata, Nishimura, Eto, & Nagata, 2015; Suzuki, Umeda, Sims, & Nagata, 2010; Yang et al., 2012). TMEM16F is not expressed in patients with Scott Syndrome, who show deficient Ca^2+^‐dependent phosphatidylserine exposure in platelets and other blood cells (Millington‐Burgess & Harper, 2019; van Geffen, Swieringa, & Heemskerk, 2016; Zwaal, Comfurius, & Bevers, 2004). Similarly, platelets from *Tmem16f* ^*−/−*^ mice show reduced phosphatidylserine exposure (Yang et al., 2012). *Tmem16f* ^*−/−*^ mice showed no occlusion in a carotid artery thrombosis model (Yang et al., 2012) and platelet‐specific conditional *Tmem16* ^*ffl/fl‐PFCre*^ mice showed prolonged occlusion time compared to wild‐type mice (Baig et al., 2016; Fujii et al., 2015). This indicates that TMEM16F on platelets (and other cells) is a key regulator of occlusive thrombosis and loss of TMEM16F limits thrombosis. TMEM16F may therefore be a potential new antithrombotic target. A selective inhibitor of TMEM16F is required to test this concept.

R5421 (ethaninidothioic acid) was originally reported as an inhibitor of platelet scramblase activity (Dekkers et al., 1998). R5421 has been widely used by other groups to determine the role of scramblase activity in other cells and systems including erythrocytes, viral infection and placental function (Berghold et al., 2015; Wesseling et al., 2016; Younan et al., 2018). No other selective inhibitor of scramblase activity is currently available.

Despite being used as a scramblase inhibitor in different systems, the specificity of R5421 has not been well characterised. Here, we sought to determine whether R5421 selectively inhibits platelet scramblase function, which would indicate that it might be a suitable scaffold for the development of TMEM16F inhibitors. However, we show that R5421 has multiple off‐target inhibitory effects in platelets, including disruption of Ca^2+^ signalling and inhibition of αIIbβ_3_ activation, undermining its use as a selective scramblase inhibitor. Furthermore, similar properties were seen with the related molecule, thiodicarb, a widely used pesticide.

## 2 METHODS

### 2.1 Washed platelet preparation

Blood was taken by venepuncture from healthy, drug‐free volunteers who had given informed, written consent in accordance with the Declaration of Helsinki and with approval from the Human Biology Research Ethics Committee, University of Cambridge.

Blood was drawn into sodium citrate (3.2% v/v) vacutainers. Acid citrate dextrose (ACD; 85‐mM tri‐sodium citrate, 71‐mM citric acid, 111‐mM d‐glucose) was added (1:7 v/v) and PRP was separated by centrifugation (200 *g*, 10 min, ambient temperature and without any brake).

Washed platelets were prepared as previously described (Wei, Malcor, & Harper, 2018). PRP was collected and diluted 1:1 with HEPES‐buffered saline (HBS) (in mM: 135 NaCl, 3 KCl, 10 HEPES, 1 MgCl_2_.6H_2_O, 0.34 Na_2_HPO_4_, 12 NaHCO_3_; pH 7.4) supplemented with 5‐mM d‐glucose (HBS‐glucose); 100‐nM PGE_1_ and apyrase (grade VII; 0.02 U·ml^−1^) were added to prevent platelet activation. Platelets were pelleted by centrifugation (600 *g*, 10 min, ambient temperature). The platelet pellet was resuspended in HBS‐glucose at 5 × 10^7^ platelets·ml^−1^ unless otherwise specified. Apyrase was added (0.02 U·ml^−1^) and platelets were rested in a 30°C water bath for 30 min before use. Prior to use, CaCl_2_ was added so stimulations occurred at 2‐mM Ca^2+^.

### 2.2 Flow cytometry

Platelets were pretreated with R5421, screening compounds A1–A16 or 0.1% DMSO as vehicle control at temperatures and for durations specified in the main text in the presence of 2‐mM Ca^2+^. Platelets were stimulated with agonists for durations and at temperatures as specified in the main text. Following stimulations, platelets were stained with fluorescently conjugated antibodies and proteins for 2 min at ambient temperature, then fixed with 1% paraformaldehyde (PFA) and diluted with HBS for analysis by flow cytometry (BD Accuri C6). Phycoerythrin (PE)‐Cy7‐conjugated anti‐CD41a antibody [AB_2573348] (1:100) was used to positively identify platelets. Annexin V‐FITC (1:100 in 2.5‐mM Ca^2+^ HBS‐glucose) was used to detect surface phosphatidylserine exposure. α_IIb_β_3_ integrin activation was measured using FITC‐conjugated PAC‐1 antibody [AB_2745523] (1:20). P‐selectin surface exposure was measured using phycoerythrin‐conjugated anti‐CD62P antibody [AB_10668715] (1:20) as a marker of α‐granule release. To measure surface expression of glycoprotein VI, Alexa 647‐conjugated anti‐ glycoprotein VI antibody [AB_2738900] was used. For endpoint analysis, 20,000 events were collected. Compensation was performed when necessary using OneComp eBeads according to the manufacturer's instructions.

### 2.3 Plate‐based annexin V binding assay

This assay was performed in white flat‐bottomed 96‐well plates; 90‐μl platelet suspension at 1 × 10^8^ per millilitre was pre‐incubated with R5421, screening compounds A1–A16 or 0.1% DMSO as a vehicle control for 10 min in the presence of 2‐mM Ca^2+^ and then stimulated using 10‐μM A23187 (dispensed via on‐board injectors) to make a final volume of 100 μl, all at 37°C. Promega RealTime‐Glo™ Annexin V Apoptosis Detection Assay kit was used according to manufacturer's instructions. Detection buffer containing 1 × Annexin V‐SmBiT Reagent, Annexin V‐LgBiT Reagent and Annexin V NanoBiT Substrate diluted in HBS‐glucose was added in equal volume (100 μl, and luminescence was detected every 10 s using a FLUOstar Omega plate reader (BMG Labtech). The luminescence of an unstimulated control sample was subtracted from the stimulated samples to account for small increase in baseline luminescence over time. Peak scrambling rates were calculated from the gradients of luminescence traces using GraphPad Prism v.8.

### 2.4 Cal520 fluorescent signals

Platelets were loaded with Cal520 by incubating undiluted PRP with 1‐μM Cal520/AM for 10 min at 30°C. Apyrase (0.02 U·ml^−1^) and PGE_1_ (100 nM) were present throughout. PRP was diluted 1:1 with HBS‐glucose and excess dye was washed away during the second centrifugation step, as above. Platelets were resuspended at 5 × 10^7^ platelets·ml^−1^ in HBS‐glucose and apyrase (0.02 U·ml^−1^) was added and rested in a water bath at 30°C for 30 min before use. Cal520‐loaded platelets were incubated with compounds for the times and at temperatures described in the main text in the presence of 2‐mM Ca^2+^. Cal520 fluorescence was monitored at ambient temperature using a FLUOStar Omega Plate Reader (BMG Labtech; excitation: 485 nm, emission: 520 nm) in black, flat‐bottomed 96‐well plates. Fluorescence in unstimulated Cal520‐loaded platelets was measured for 1 min before stimulation with the indicated agonist (dispensed via the FLUOStar Omega reagent injectors) and fluorescence was measured every 5 s for a further 10 min. Fluorescence values were normalised to initial fluorescence values of vehicle controls containing 0.1% DMSO. This F/F_0_ normalisation accounts for variation in Cal520 loading between different experiments. AUC represents the sum of fluorescence readings above baseline following stimulation.

### 2.5 In silico ligand‐based screen

The 3D structure of R5421 was obtained from PubChem (ID: 124220090) and was then energy‐minimised using MMFF94 forcefield implemented in Open Babel 3.0 (O'Boyle et al., 2011). Using the energy‐minimised R5421 as a query, we screened a subset of the Molport database comprising 10,000 readily purchasable diverse chemical scaffolds, using ROCS (Rapid Overlay of Chemical Structures, version 3.3.1, OpenEye Scientific Software. Santa Fe, NM, USA) (Rush, Grant, Mosyak, & Nicholls, 2005). The top 100 hits were ranked based on Tanimoto combo scores and we purchased the top 16 molecules for wet testing. All compounds were screened at 100 μM and 0.1% DMSO used as a vehicle control. Alignments of compounds and similarity scores to R5421 were calculated using Forge software (Forge, version 10.6, Cresset) (Cheeseright, Mackey, Rose, & Vinter, 2006).

### 2.6 Statistics and data analysis

The number of biological repeats in each experiment is 5 and represents independent platelet preparations from different donors. Statistical analyses were performed in GraphPad Prism v.8. All statistical tests were carried out using matched analyses of independent values (not technical replicates). Where individual treatment conditions were compared to DMSO controls, two‐tailed Student's paired *t*‐tests were used. Where multiple treatment conditions were compared to DMSO controls, data were analysed using two‐way ANOVAs, both ways matching and Sidak's multiple comparison tests as appropriate (and only when *F* in ANOVA achieved statistical significance). Homogeneity of variance was tested using Levene's test (in Excel). *P* values <0.05 were taken to be statistically significant (Curtis et al., 2015). The data and statistical analysis comply with the recommendations of the *British Journal of Pharmacology* on experimental design and analysis in pharmacology.

### 2.7 Materials

R5421 was purchased from Endotherm Life Science Molecules (Saarbrücken, Germany) and dissolved in DMSO. Recombinant human (Rh) Annexin‐V FITC and OneComp eBeads were from Invitrogen, Thermo Fisher Scientific, UK. FITC Mouse Anti‐Human PAC‐1 (340507) and phycoerythrin Mouse Anti‐Human CD62P (555524) were from BD Biosciences. Adenosine 5′‐diphosphate (ADP; 01905), adrenaline hydrochloride (E4642), A23187 (C7522) and bovine thrombin (T4648) were from Sigma‐Aldrich (Poole, UK). Cal‐520 AM (21130‐AAT) was from Stratech. Vacuette tubes (9‐ml 9NC Coagulation sodium citrate 3.2%) were from Grenier Bio‐One. PAR1‐activating peptide (AP) (SFLLRN‐amide trifluoroacetate salt; 4031274) was from Bachem. RealTime‐Glo™ Annexin V Apoptosis Assay (JA1001) was from Promega. Screening compounds A1–A16 were purchased via Molport (see Table S1 for further details).

Cross‐linked collagen‐related peptide (CRP‐XL) was synthesised by Dr Bonna according to previously published methods (Morton, Hargreaves, Farndale, Young, & Barnes, 1995).

### 2.8 Nomenclature of targets and ligands

Key protein targets and ligands in this article are hyperlinked to corresponding entries in http://www.guidetopharmacology.org, the common portal for data from the IUPHAR/BPS Guide to PHARMACOLOGY (Harding et al., 2018), and are permanently archived in the Concise Guide to PHARMACOLOGY 2019/20 (Alexander et al., 2019).

## 3 RESULTS

### 3.1 Ethaninidothioic acid (R5421) inhibits platelet phosphatidylserine exposure in response to A23187 or thrombin and CRP‐XL

Consistent with original reports, R5421 inhibited platelet phosphatidylserine exposure following stimulation with calcium ionophore, A23187 (10 μM) in a time‐ and temperature‐dependent manner.

**FIGURE 1:**
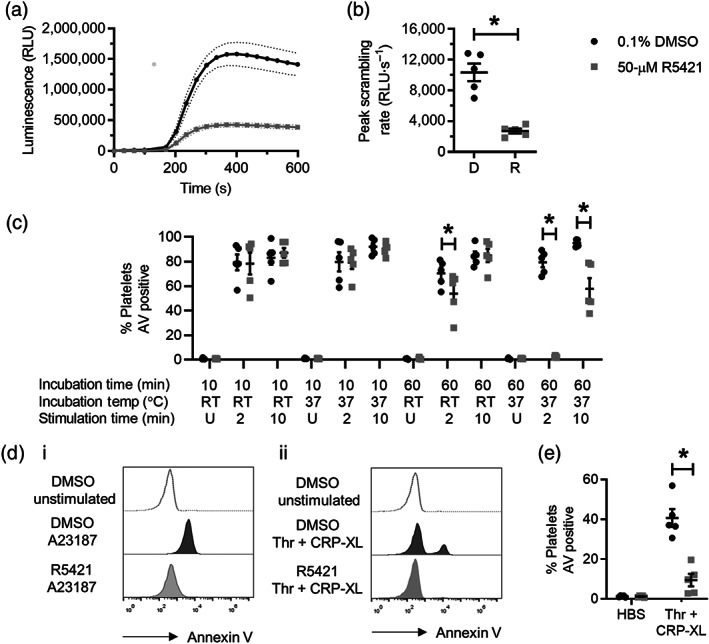
R5421 inhibits platelet phosphatidylserine (PS) exposure in response to A23187 or thrombin + CRP‐XL. (a) Luminescence traces from a real‐time plate‐based annexin V binding assay following stimulation with 10‐μM A23187. Incubations with R5421 or DMSO (as vehicle control) prior to stimulation were for 10 min at 37°C. RLU, relative luminescence units. Data are mean ± SEM (dotted lines; *n* = 5). (b) Peak scrambling rate from plate‐based annexin V binding assay. Mean ± SEM is indicated (*n* = 5; *P < 0.05). (c) Flow cytometry annexin V (AV) binding after stimulation with 10‐μM A23187 following different incubation conditions with ethaninidothioic acid (R5421) or DMSO (10‐ or 60‐min incubations at ambient temperature [RT] or 37°C) and stimulation times (2 or 10 min; U, unstimulated). All stimulations were at ambient temperature. Black dots: 0.1% DMSO. Grey squares: 50‐μM R5421 (*n* = 5; *P < 0.05). (d) Representative histograms of AV binding measured by flow cytometry following stimulation with 10‐μM A23187 or 1 U thrombin + 1 μg·ml^−1^ CRP‐XL. Unshaded: unstimulated platelets. Black shading: 0.1% DMSO‐treated platelets. Grey shading: 50‐μM R5421‐treated platelets. (e) Flow cytometry annexin V binding after a 10‐min incubation with R5421 or DMSO at ambient temperature and a 10‐min stimulation with 1 U thrombin + 1 μg·ml^−1^ CRP‐XL at ambient temperature. Black dots: 0.1% DMSO. Grey squares: 50‐μM R5421 (*n* = 5; *P < 0.05)

Two assays were used to demonstrate this inhibition. The first was a luminescence‐based assay using two annexin V fusion proteins containing complementary subunits of NanoBiT Luciferase. Phosphatidylserine exposure allows the annexin V fusion proteins to bind in proximity on the plasma membrane and generate a luminescence signal. This assay provides a measure of the rate of platelet phosphatidylserine exposure. The peak rate of phosphatidylserine exposure was slowed following incubation with R5421 for 10 min at 37°C compared with DMSO (Figure 1a,b). The mean pIC_50_ for inhibition of peak scrambling rate by R5421 was 4.81 ± 0.06 (*n* = 5; Table 1).

**TABLE 1:** pIC_50_ for inhibition of platelet functions by ethaninidothioic acid (R5421) and thiodicarb (A1)

Assay	R5421	Thiodicarb (A1)
Luminescence annexin V binding assay (plate reader) Drug incubation: 10 min, 37°C Stimulation: 10‐μM A23187, 37°C	4.81 ± 0.06	Not determined
Annexin V % positive (flow cytometry) Drug incubation: 60 min, 37°C Stimulation: 10‐μM A23187, 2 min, 37°C	4.41 ± 0.04	Not determined
Annexin V % positive (flow cytometry) Drug incubation: 10 min, RT Stimulation: 1 U·ml^−1^ thrombin + 1 μg·ml^−1^ CRP‐XL, 10‐min stimulation, RT	5.01 ± 0.40	3.74 ± 0.49
PAC‐1 binding (flow cytometry) Drug incubation: 10 min, RT Stimulation: 10‐μM PAR1‐AP, 10 min, RT	5.32 ± 0.09	5.03 ± 0.27
CD62P surface expression (flow cytometry) Drug incubation: 10 min, RT Stimulation: 10‐μM PAR1‐AP, 10 min, RT	4.67 ± 0.06	4.52 ± 0.12

*Note*: pIC_50_ data are given as mean ± SEM.

Abbreviation: RT, room temperature.

Platelet phosphatidylserine exposure was also measured by endpoint flow cytometry at 2 and 10 min post stimulation with A23187 (10 μM). In contrast to the luminescence‐based assay, which monitors phosphatidylserine exposure rate, 10‐min incubation with R5421 did not affect endpoint annexin V binding measured by flow cytometry under any conditions tested. A 60‐min incubation of R5421 at both ambient temperature and 37°C inhibited phosphatidylserine exposure at 2 min following stimulation. A 60‐min incubation of R5421 at 37°C also inhibited phosphatidylserine exposure at 10 min following stimulation (Figure 1c). Maximal endpoint inhibition was seen following incubation with R5421 for 60 min at 37°C and following 2 min of stimulation, consistent with an inhibition of the rate of phosphatidylserine exposure as more platelets become phosphatidylserine positive by 10 min post stimulation.

R5421 also inhibited endpoint platelet phosphatidylserine exposure following a 10 min stimulation with thrombin and CRP‐XL, a combination of agonists required for a substantial percentage of platelets to expose phosphatidylserine (Harper et al., 2013). Surprisingly, this inhibition was achieved following only a 10‐min incubation at ambient temperature (Figure 1d,e), despite R5421 having no effect on A23187‐induced phosphatidylserine exposure. This suggests that the mechanism of inhibition of phosphatidylserine exposure in response to the two different stimuli may be different.

### 3.2 R5421 inhibits platelet αIIbβ_3_ integrin activation and α‐granule release following stimulation with PAR1‐AP

**FIGURE 2:**
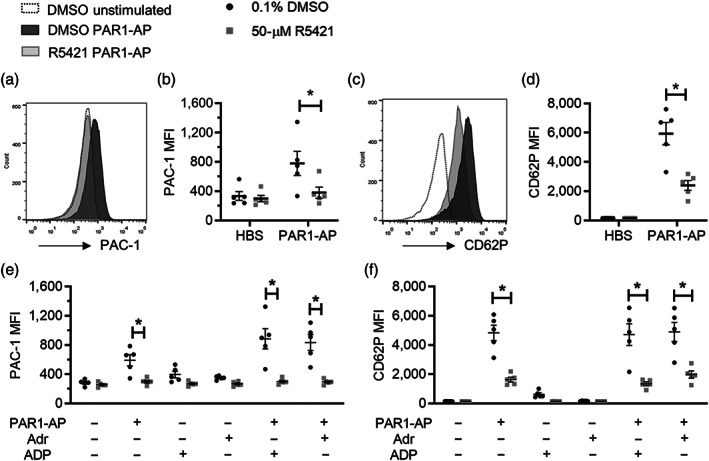
R5421 inhibits platelet integrin α_IIb_β_3_ activation and α‐granule release following stimulation with PAR1‐AP. Platelets were treated with ethaninidothioic acid (R5421; 50 μM) or DMSO (0.1%; vehicle control) for 10 min (ambient temperature) prior to stimulation for 10 min. α_IIb_β_3_ activation was measured by flow cytometry as PAC‐1 binding (FITC conjugated). α‐granule secretion was measured by anti‐CD62P (P‐selectin) antibody binding (phycoerythrin (PE) conjugated). (a) Representative histograms of PAC‐1 binding. (b) PAC‐1‐FITC median fluorescence intensity (MFI) following stimulation with 10‐μM PAR1‐AP (*n* = 5; *P < 0.05). (c) Representative histograms of anti‐CD62P‐PE fluorescence. (d) CD62P‐PE MFI following stimulation with 10‐μM PAR1‐AP (*n* = 5; *P < 0.05). (e) PAC‐1 binding following stimulation with 10‐μM PAR1‐AP ± 10‐μM ADP or 10‐μM adrenaline. (f) CD62P binding following stimulation with PAR1‐AP ± 10‐μM ADP or 10‐μM adrenaline. Black dots: 0.1% DMSO. Grey squares: 50‐μM R5421. Individual data are given together with mean ± SEM (*n* = 5; *P < 0.05)

To determine whether R5421 has off‐target effects in platelets, R5421‐treated platelets were stimulated with proteinase‐activated receptor 1‐activating peptide (PAR1‐AP; 10 μM). This stimulus was chosen as it effectively stimulates platelets to become pro‐aggregatory but very few become pro‐coagulant (Harper & Poole, 2011). R5421 inhibited αIIbβ_3_ integrin activation, measured by PAC‐1 binding (Figure 2a,b), and α‐granule release, measured by an anti‐CD62P antibody (Figure 2c,d). The pIC_50_ values for these inhibitions are shown in Table 1. These inhibitions were observed after a 10‐min incubation with R5421 at ambient temperature, conditions under which R5421 did not inhibit A23187‐induced phosphatidylserine exposure (Figure 1). R5421 inhibited αIIbβ_3_ activation or α‐granule secretion even when platelets were co‐stimulated with PAR1‐AP and ADP (10 μM), suggesting that these inhibitions were not a consequence of reduced dense granule secretion (Harper, van den Bosch, Hers, & Poole, 2015). Similarly, R5421 also inhibited αIIbβ_3_ activation or α‐granule secretion when platelets were co‐stimulated with PAR1‐AP and adrenaline (10 μM) to bypass P2Y_12_, suggesting that R5421 does not directly inhibit P2Y_12_ (Figure 2e,f).

### 3.3 R5421 inhibits platelet Ca^2+^ signalling in response to a range of agonists

**FIGURE 3:**
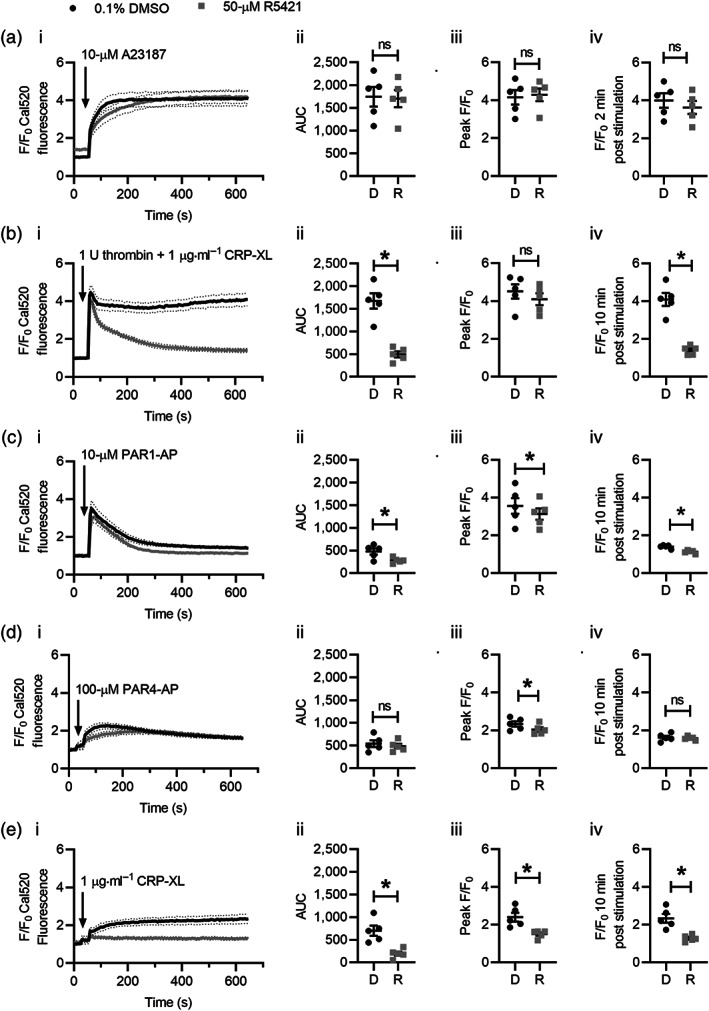
R5421 inhibits platelet Ca^2+^ signalling in response to a range of agonists. Platelets were loaded with the Ca^2+^‐sensitive fluorescence dye, Cal520. Fluorescence values were normalised to the initial fluorescence values of vehicle controls containing 0.1% DMSO in matched samples to account for variation in dye loading (F/F_0_). Platelets were treated with ethaninidothioic acid (R5421; 50 μM; grey lines and dots) or DMSO (0.1%; black lines and squares) under conditions that inhibited phosphatidylserine (PS) exposure in previous experiments (i.e., in (a), 60‐min incubation at 37°C and in (b)–(d), 10‐min incubation at ambient temperature). Where indicated, platelets were stimulated with (a) A23187 (10 μM); (b) 1 U thrombin + 1 μg·ml^−1^ CRP‐XL; (c) 10‐μM PAR1‐AP; (d) 100‐μM PAR4‐AP; and (e) 1 μg·ml^−1^ CRP‐XL. All stimulations at ambient temperature. Data are presented as (i) F/F_0_ Cal520 fluorescence following stimulation; (ii) AUC above baseline (F/F_0_ = 1); (iii) peak F/F_0_ following stimulation; and (iv) F/F_0_ at corresponding time of inhibition of PS exposure in flow cytometry (i.e., in (a), 2 min and in (b)–(e), 10 min). In (ii)–(iv) for each stimulation, dots and squares represent independent experiments. The mean ± SEM is also given. *n* = 5 for all experiments; *P < 0.05

To investigate whether R5421 affects platelet Ca^2+^ signalling, platelets were loaded with the Ca^2+^‐sensitive fluorescent dye, Cal520. Stimulation with A23187 resulted in a rapid and sustained increase in Cal520 fluorescence, which was not significantly affected by R5421 pretreatment (Figure 3a). In contrast, R5421 inhibited Cal520 fluorescence following co‐stimulation with thrombin + CRP‐XL. Although the peak F/F_0_ was not significantly different, R5421 inhibited the sustained Ca^2+^ signal, whether quantified by AUC or F/F_0_ 10 min post stimulation (Figure 3b).

R5421 inhibited platelet Cal520 fluorescence following stimulation with PAR1‐AP, although the magnitude of this effect was relatively small (Figure 3c). Similarly, although R5421 partially inhibited the peak F/F_0_ following stimulation with the PAR4‐AP, it did not significantly affect the sustained signal, quantified by AUC or F/F_0_ at 10 min post stimulation (Figure 3d). In contrast, R5421 significantly inhibited the Cal520 fluorescence signal in response to the glycoprotein VI agonist, CRP‐XL. Both the peak and sustained signals were significantly inhibited (Figure 3e). R5421 did not induce shedding of glycoprotein VI from the platelet surface (Figure S1).

These data indicate that R5421 inhibits cytosolic Ca^2+^ signalling, particularly through the glycoprotein VI pathway. This is likely to account for the inhibition of phosphatidylserine exposure under conditions where the scramblase is not itself inhibited.

### 3.4 Thiodicarb inhibits platelet phosphatidylserine exposure, αIIbβ_3_ integrin activation and α‐granule release

**FIGURE 4:**
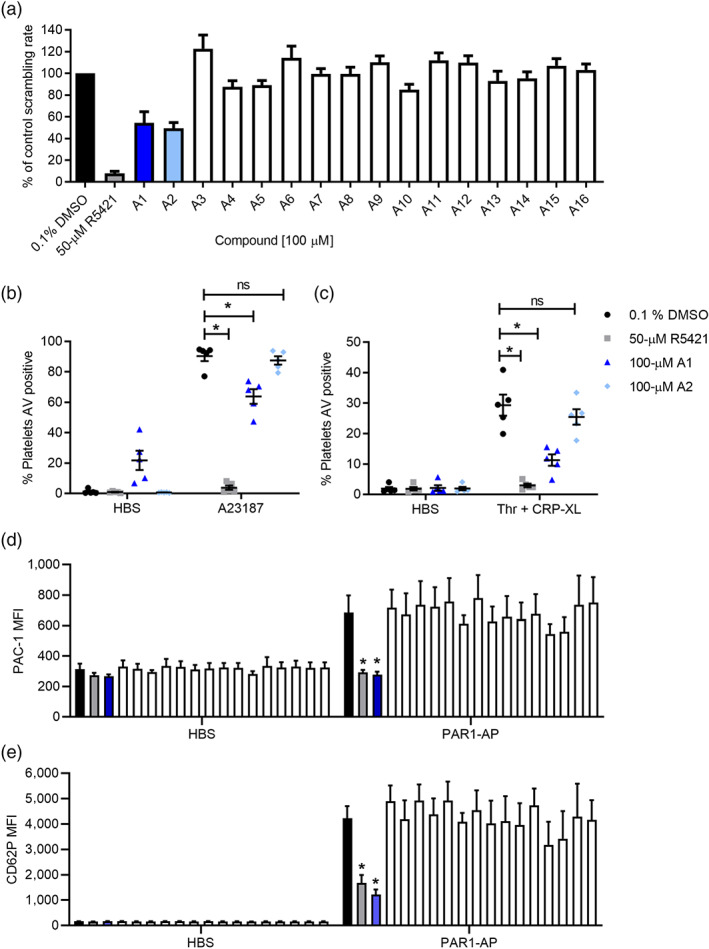
A1 (thiodicarb) inhibits platelet phosphatidylserine (PS) exposure, α_IIb_β_3_ integrin activation and α‐granule release. (a) Maximum scrambling rate from plate‐based annexin V binding assay of 16 compounds (A1–A16; each 100 μM; 60 min, 37°C) identified by in an in silico ligand similarity screen, compared to platelets treated with ethaninidothioic acid (R5421) or DMSO (mean + SEM is shown; *n* = 5). (b) Percentage of platelets binding annexin V (AV) after stimulation with 10‐μM A23187 (10 μM, 2 min; ambient temperature), measured by flow cytometry (*n* = 5; *P < 0.05). (c) Platelets were treated with DMSO, R5421, A1 or A2 (10 min, ambient temperature). The percentage of platelets binding annexin V (AV) after stimulation with 1 U thrombin + 1 μg·ml^−1^ CRP‐XL (10 min, ambient temperature) measured by flow cytometry is shown. Black dots: 0.1% DMSO. Grey dots: 50‐μM R5421. Dark blue triangles: 100‐μM A1 (thiodicarb). Light blue diamonds: 100‐μM A2 (*n* = 5). (d) PAC‐1 binding following stimulation with 10‐μM PAR1‐AP (or with HBS as vehicle control). (e) Anti‐CD62P antibody binding following stimulation with 10‐μM PAR1‐AP. For (d) and (e), 10‐min incubation with DMSO, R5421, A1 or A2 at ambient temperature and 10‐min stimulation at ambient temperature; *n* = 5

Since R5421 appeared to have multiple inhibitory effects on platelets, we investigated whether it was possible to pharmacologically separate these effects by using molecules similar to R5421. An in silico structure‐based screen around R5421 identified 16 commercially available compounds that we tested in our plate‐based annexin V binding assay. The structures are given in Table S1 alongside compound similarity scores, accounting for fields and shape, to R5421 generated using Forge v10.6 (Cresset). Compounds A1 and A2 inhibited the rate of scrambling compared to vehicle‐treated control following a 10‐min incubation at 37°C (Figure 4a). We then tested these compounds in our flow cytometry‐based assay, to exclude any inhibition due to assay‐dependent effects. Only compound A1 also inhibited platelet phosphatidylserine exposure in response to a 2‐min stimulation with A23187 following a 60‐min incubation at 37°C. Under these incubation conditions, A1 treatment also tended towards increased phosphatidylserine exposure in unstimulated platelets. A1 also inhibited phosphatidylserine exposure following a 10‐min incubation at ambient temperature and a 10‐min stimulation with thrombin + CRP‐XL when measured by flow cytometry (Figure 4b,c). Unfortunately, A1 also inhibited platelet αIIbβ_3_ integrin activation and α‐granule release following stimulation with PAR1‐AP (Figure 4d,e; Table 1). When the compounds were unblinded, A1 was revealed to be thiodicarb, a pesticide.

### 3.5 Thiodicarb inhibits platelet Ca^2+^ signalling in response to a range of agonists

**FIGURE 5:**
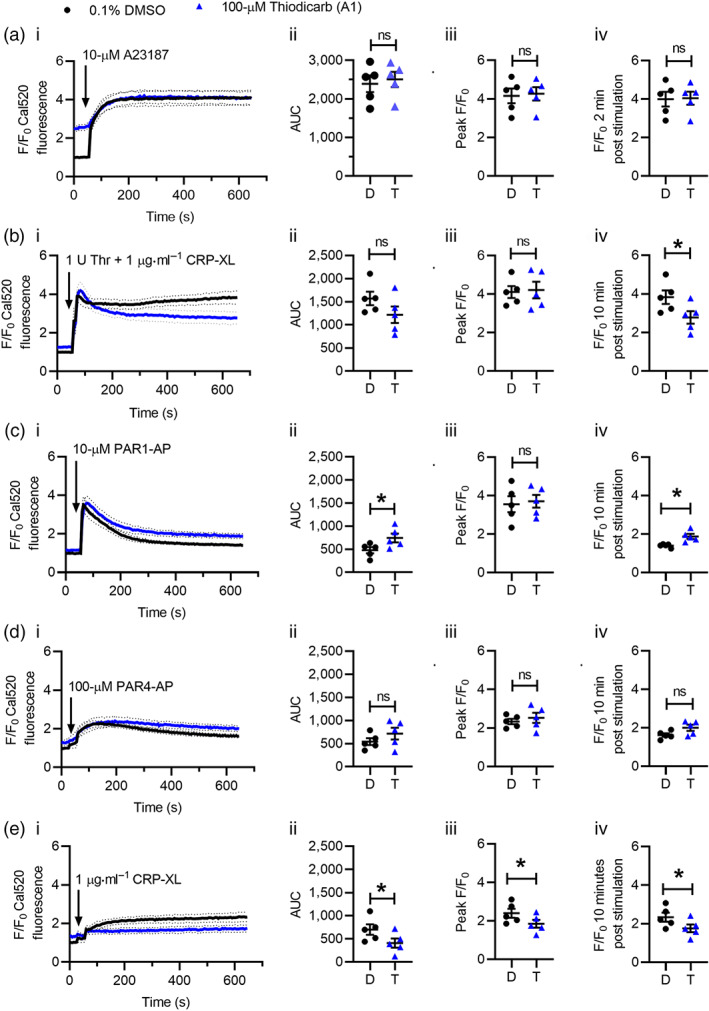
Thiodicarb inhibits platelet Ca^2+^ signalling in response to a range of agonists. Ca^2+^ signalling was monitored by Cal520 fluorescence as in Figure 3. Cal520‐loaded platelets were treated with thiodicarb under conditions that inhibited phosphatidylserine (PS) exposure in previous experiments (i.e. in (a), 60‐min incubation at 37°C and in (b)–(d), 10‐min incubation at ambient temperature). Where indicated by the arrows, platelets were stimulated with (a) 10‐μM A23187; (b) 1 U thrombin + 1 μg·ml^−1^ CRP‐XL; (c) 10‐μM PAR1‐AP; (d) 100‐μM PAR4‐AP; and (e) 1 μg·ml^−1^ CRP‐XL. All stimulations at ambient temperature. Data are presented as (i) F/F_0_ Cal520 fluorescence following stimulation; (ii) AUC above baseline (F/F_0_ = 1); (iii) peak F/F_0_ following stimulation; and (iv) F/F_0_ at corresponding time of inhibition of PS exposure in flow cytometry (i.e., in (a), 2 min and in (b)–(e), 10 min). In (ii)–(iv) for each stimulation, dots and triangles represent independent experiments. The mean ± SEM is also given. Black dots and traces: 0.1% DMSO. Blue triangles and traces: 100‐μM thiodicarb. *n* = 5 for all experiments; *P < 0.05

Thiodicarb did not affect Cal520 fluorescence following stimulation with A23187 (quantified as AUC, peak F/F_0_ or F/_0_ at 2 min following stimulation; Figure 5a). However, we did note the increased Cal520 fluorescence at the beginning of our readings following a 60‐min incubation of thiodicarb at 37°C (Figure 5a, i). Addition of thiodicarb to Cal520‐loaded platelets caused a slow, sustained increased in fluorescence (Figure S2), suggesting disruption of Ca^2+^ homeostasis. This was not seen with R5421.

Thiodicarb (10‐min incubation) inhibited Cal520 fluorescence following stimulation with thrombin + CRP‐XL. As with R5421, although the peak F/F_0_ was unaffected, the sustained signal was reduced (Figure 5b). The reduction in the sustained signal was less pronounced than seen with R5421, which may reflect the additional disruption of Ca^2+^ homeostasis caused by thiodicarb (Figure S2). In contrast, Cal520 fluorescence following stimulation with PAR1‐AP or PAR4‐AP was not inhibited (Figure 5c,d). Rather, there was a small increase in the PAR1‐AP‐stimulated signal. Consistent with R5421, thiodicarb inhibited Cal520 fluorescence following stimulation with glycoprotein VI quantified by AUC, peak F/F_0_, and F/F_0_ at 10 min post stimulation (Figure 5e). As with R5421, thiodicarb did not induce glycoprotein VI shedding (Figure S1). These data indicate that thiodicarb also inhibits cytosolic Ca^2+^ signalling, particularly through the glycoprotein VI pathway, although there is additional disruption of Ca^2+^ homeostasis in unstimulated platelets following prolonged treatment with thiodicarb.

## 4 DISCUSSION

Ethaninidothioic acid (R5421) was first described as an inhibitor of scramblase activity in 1998 (Dekkers et al., 1998) and has been used to investigate the roles of phospholipid scrambling in several models (Berghold et al., 2015; Wesseling et al., 2016; Younan et al., 2018). R5421 might therefore provide a useful scaffold for further development of potent and selective TMEM16F inhibitors. However, the pharmacology of R5421 has not been previously thoroughly characterised, and here, we report multiple off‐target effects of R5421 in platelets. In addition to inhibition of phospholipid scrambling, R5421 disrupts Ca^2+^ signalling, particularly downstream of glycoprotein VI. R5421 additionally inhibits platelet αIIbβ_3_ integrin activation and α‐granule release, an effect that is unlikely to be related to inhibition of scramblase activity or intracellular Ca^2+^ signalling. These off‐target effects of R5421 on platelet function make R5421 an unsuitable compound to assess the role of phospholipid scrambling in platelet function and thrombosis and question its use in other systems.

We have confirmed that R5421 directly inhibits platelet scramblase activity. Using the calcium ionophore A23187 to stimulate platelet phosphatidylserine exposure, we bypassed the receptors and intracellular signalling required for phosphatidylserine exposure using physiological agonists and directly raised intracellular [Ca^2+^] to activate the scramblase protein, TMEM16F. R5421 inhibited platelet phosphatidylserine exposure following A23187 stimulation in a time‐ and temperature‐dependent manner. R5421 did not affect A23187‐induced intracellular Ca^2+^ signals under the same incubation conditions that led to inhibition of A23187‐induced phosphatidylserine exposure. Therefore, R5421 does not interfere with the calcium ionophore actions of A23187 but has a direct effect on scramblase activity.

Maximum inhibition was achieved following a 60‐min incubation of 50‐μM R5421 at 37°C. This is consistent with the original report (Dekkers et al., 1998), who found a similar time dependency to the inhibition. They also reported that the inhibition was not readily reversed by washing the platelets, suggesting that the action of R5421 is either irreversible or has a very slow off‐rate. R5421 slowed the rate of phosphatidylserine exposure. This was demonstrated in our plate‐based assay. Similarly, in our endpoint flow cytometry, R5421 completely inhibited annexin V binding at 2 min following stimulation. In contrast, more platelets become annexin V‐positive by 10 min, as detected by flow cytometry, leading to an apparent difference between the results of our two annexin V binding assays. One possible explanation is that the plate‐based assay may require a greater density of phosphatidylserine on platelet surface. The plate‐based assay requires two different conjugated annexin Vs (Annexin V‐SmBiT and Annexin V‐LgBiT) to combine in proximity to generate a luminescence signal, which, we suggest, requires high cell surface density of phosphatidylserine to be effective. In contrast, the flow cytometer requires only a single annexin V‐FITC to bind to the platelet to generate a signal. The signal strength of the flow cytometer assay is therefore likely to be less dependent on the phosphatidylserine surface density of a single cell. Notably, a time‐resolved FRET assay of annexin V binding showed a similar lower sensitivity compared to flow cytometry; this TR‐FRET assay also required two differently labelled annexin V monomers to oligomerise to generate a signal (Gasser, Hehl, & Millward, 2009).

Moreover, annexin V oligerimises into a 2D lattice when it binds to a phosphatidylserine‐exposing membrane. Annexin V‐FITC oligomerisation on binding to a phosphatidylserine‐exposing membrane may result in a fluorescence signal that is disproportionate to the phosphatidylserine surface density. Flow cytometry analysis of platelet phosphatidylserine exposure often results in an all‐or‐nothing response, with platelets characterised as positive or negative for annexin V binding (Abbasian, Millington‐Burgess, Chabra, Malcor, & Harper, 2020; Harper et al., 2013). The annexin V‐positive platelets have the same high fluorescence. This is how we have interpreted our flow cytometry data in this study. The detection of annexin V binding to phosphatidylserine using flow cytometry rapidly saturates with small increases in the percentage of phosphatidylserine exposed in the membrane, as shown by experiments with bilayers of defined composition adsorbed to glass beads (Stuart, Reutelingsperger, & Frederik, 1998). Similarly, the fluorescence of annexin V‐FITC bound to liposomes of varying phosphatidylserine percentage showed an all‐or‐nothing response above a threshold percentage of phosphatidylserine (Shi et al., 2006). Lactadherin‐FITC, which does not oligomerise, gave a graded fluorescence signal that was proportional to the phosphatidylserine percentage.

The two‐part annexin V detection system (as in the luminescence assay here or the TR‐FRET assay described above) may mean that the signal is less likely to be saturated by small increases in the surface density of phosphatidylserine and may give a signal that is more proportional to the degree of phosphatidylserine exposure, as oligomerisation is required for its signal. In both cases, partial inhibition of phosphatidylserine exposure may be sufficient to show inhibition in the luminescence assay, but not sufficient to reduce the phosphatidylserine exposure below the all‐or‐nothing threshold for the flow cytometry assay. This suggests that almost complete inhibition of TMEM16F may be required to see an inhibitory effect in flow cytometry. In contrast, a potential benefit of the luminescence‐based assay is that it can be used to detect drugs that reduce the rate of scrambling even when endpoint flow cytometry does not show a difference.

Complete inhibition of TMEM16F may be required for a therapeutic benefit, however. In an unstimulated platelet, phosphatidylserine that becomes exposed on the surface is transported back into the inner leaflet by an aminophospholipid translocase (“flippase”). This flippase activity is inhibited in procoagulant platelets, meaning that phosphatidylserine exposure by TMEM16F is unopposed. If the rate of scrambling is substantially reduced but not abolished, phosphatidylserine will still eventually accumulate in the outer leaflet of the membrane. A potential example is compound A2 from the ligand‐based screen (discussed below).

R5421 inhibited platelet phosphatidylserine exposure following stimulation with thrombin + CRP‐XL, but with a different time and temperature dependency to following A23187. Complete inhibition of phosphatidylserine exposure was observed by flow cytometry following a 10‐min incubation with R5421 at room temperature. Notably, under these conditions, there was no inhibition of A23187‐induced phosphatidylserine exposure in either assay. This suggested that following thrombin + CRP‐XL stimulation R5421 might be inhibiting platelet phosphatidylserine exposure through a different mechanism. Since phosphatidylserine exposure is dependent on intracellular Ca^2+^ signalling, we investigated whether this was affected by R5421. R5421 significantly inhibited cytosolic Ca^2+^ signalling following thrombin + CRP‐XL stimulation. Notably, although the initial peak increase was unaffected, the sustained Ca^2+^ signal was almost completely inhibited. Since the increase in intracellular Ca^2+^ concentration must be sustained to trigger phosphatidylserine exposure (Keuren et al., 2005), the inhibition of phosphatidylserine exposure by R5421 under these conditions is likely to be due to inhibition of Ca^2+^ signalling rather than direct inhibition of the scramblase.

To further investigate the effect of R5421 on intracellular Ca^2+^ signalling, we stimulated platelets separately with agonists of PAR1, PAR4, and glycoprotein VI, the main receptors activated during stimulation with thrombin + CRP‐XL. There were some small effects on Ca^2+^ signalling downstream of PAR1 and PAR4, though these are unlikely to account for the large inhibition of Ca^2+^ signalling described above. In contrast, Ca^2+^ signalling downstream of glycoprotein VI (VI (GPVI) was almost completely abolished. The signalling between receptor and Ca^2+^ signal is notably different downstream of PAR1 or PAR4 and glycoprotein VI. PAR1 and PAR4 are GPCRs, predominately coupled to activation of Gαq (Gieseler, Ungefroren, Settmacher, Hollenberg, & Kaufmann, 2013) and subsequent activation of PLCβ (Nieman, 2016). Glycoprotein VI is a member of the immunoglobulin family of receptors. It is associated with an immunoreceptor tyrosine‐based activation motif (ITAM)‐containing protein, FcR γ chain (Moroi & Jung, 2004). Activation of glycoprotein VI triggers phosphorylation of the ITAM motif by Src family TKs, followed by recruitment and activation of spleen associated tyrosine kinase (Syk TK) and subsequent phosphorylation of PLCγ2 (Watson, Auger, McCARTY, & Pearce, 2005). Phosphoinositide 3‐kinases and Bruton tyrosine kinase (Btk), a Tec family TK, are also involved in regulating PLCγ2 (Quek, Bolen, & Watson, 1998; Suzuki‐Inoue, Inoue, Frampton, & Watson, 2003). Although the molecular target of R5421 is not known, the different pathways between receptor activation and PLC activation provide a potential explanation for the different effect of R5421 on Ca^2+^ signalling downstream of PAR1 or PAR4 and glycoprotein VI.

R5421 also inhibits platelet αIIbβ_3_ integrin activation and α‐granule release following stimulation of PAR1, despite having only a small effect on intracellular Ca^2+^ signalling. The inhibition in α‐granule secretion could result from decreased αIIbβ_3_ activation as outside‐in signalling through this integrin enhances α‐granule secretion (Tucker et al., 2008). The signalling pathway from PAR1 to αIIbβ_3_ activation has been intensively investigated (Stefanini & Bergmeier, 2016, 2019). Increased intracellular Ca^2+^ concentration activates a guanine nucleotide exchange factor, CalDAG‐GEFI, leading to activation of the small GTPase, Rap1b. This promotes talin binding to cytosolic regions of β_3_ and activation of the integrin. In addition, Ca^2+^ and PKC trigger ADP secretion from dense granules. ADP activates P2Y_12_, which leads to phosphoinositide 3‐kinase (PI3‐K) activity, an increase in phosphoinositide 3,4,5 trisphosphate (PIP_3_), and PIP_3_‐dependent inhibition of Rasa3, a GTPase activating protein (GAP). This inhibition allows Rap1b to remain in its active, GTP‐bound form, maintaining αIIbβ_3_ activation. R5421 could, in principle, act at any of these steps. However, as R5421 is unlikely to be solely acting to inhibit ADP release by dense granule secretion, as α_IIb_β_3_ activation could not be rescued by co‐stimulation with PAR1‐AP and ADP. Similarly, although loss of dense granule secretion reduces α‐granule secretion, this would also be rescued by co‐stimulation with ADP (Harper et al., 2015; Savage et al., 2013). In addition, R5421 is unlikely to inhibit P2Y_12_, as αIIbβ_3_ activation could not be rescued when adrenaline was used to activate α_2_‐adrenoceptors, which are coupled to Gz and can also provide PI3‐K‐dependent signalling to maintain integrin activation (Gurbel, Kuliopulos, & Tantry, 2015). Together, these data suggest that R5421 may inhibit αIIbβ_3_ activation by acting distal to receptor signalling, perhaps on Rap1b activation, though the precise target remains to be identified. Interestingly, CalDAG‐GEFI has been recently identified as a target of the antidepressant, citalopram (Roweth et al., 2018), indicating that it is possible for this protein to be inhibited by small molecules. Another possible target of R5421, a carbamate, is arylacetamide deacetylase‐like 1 (AADACL1), a lipid deacetylase that regulates Rap1b activity in platelets. Several carbamate compounds have been shown to inhibit platelet αIIbβ_3_ activation, possibly through inhibition of AADACL1 (Holly et al., 2013).

Since multiple off‐target effects of R5421 on platelet function have been identified, we sought to try and find a more selective inhibitor of platelet scramblase activity using a ligand‐based in silico screen. Sixteen compounds were identified during this screen for testing with high shape and field similarity to R5421 (similarity scores over 0.6 calculated using Forge software), and two of these, A1 and A2, were found to inhibit the rate of scrambling in our plate‐based assay. However, A1 (thiodicarb) was also effective at inhibiting platelet phosphatidylserine exposure in flow cytometry experiments following stimulation with A23187 or thrombin + CRP‐XL. This indicates that there are differences between the sensitivities of our plate‐based and flow cytometry assays, which remain to be fully understood (see above). The plate‐based assay may be a valuable approach for further investigating the mechanisms of phosphatidylserine exposure. However, as the reduction in scrambling rate was not sufficient to affect endpoint annexin V binding measured by flow cytometry, the widely used approach to measure platelet phosphatidylserine exposure, we did not continue to investigate A2 as a platelet scramblase inhibitor in this study.

As with R5421, A1/thiodicarb inhibited platelet phosphatidylserine exposure following A23187 stimulation after a 60‐min incubation at 37°C. Under these conditions, thiodicarb tends towards increasing phosphatidylserine exposure in unstimulated platelets, likely by disrupting calcium signalling and causing an increase in cytosolic [Ca^2+^]. Thiodicarb did not affect Cal520 signals following A23187 stimulation, and so we conclude that thiodicarb does have some direct effects on platelet scramblase activity. Again, similarly to R5421, thiodicarb also inhibited platelet phosphatidylserine exposure following stimulation with thrombin + CRP‐XL following a 10‐min incubation at room temperature. However, as with R5421, thiodicarb also disrupted Ca^2+^ signalling following stimulation with thrombin + CRP‐XL, indicating that we had not separated the ability to inhibit platelet scramblase activity from the effects on cytosolic Ca^2+^ signalling.

Thiodicarb is a synthetic carbamate pesticide that is extensively used for crop protection. It has been shown to have some toxicity including systemic organ damage, including occasional signs of haemorrhage and inhibition of AChE activity (Dias et al., 2013; Hoizey et al., 2008). No effects on platelets have been previously described. The effects of thiodicarb on platelet function and calcium signalling in this study may underlie some of the compound's toxicity. Whatever its targets, the toxicity of thiodicarb suggests that it, and the related R5421, may not be a suitable scaffold for the development of future scramblase inhibitors.

In conclusion, as multiple off‐target effects of R5421 have been identified, this compound is not suitable for use as a scramblase inhibitor, as it was previously described. Although a ligand‐based approach to find a more selective inhibitor of scramblase function was unsuccessful, thiodicarb was identified as a novel inhibitor of platelet scramblase activity. Thiodicarb has similar off‐target effects on platelet function as R5421 and additional disruptive effects on calcium signalling in unstimulated platelets. The multiple off‐target effects of R5421 and the inability to isolate some of these effects using ligand‐based screening approaches further suggest that R5421 is not a suitable scaffold to use in design of a selective scramblase inhibitor and other scaffolds should be investigated.

## AUTHOR CONTRIBUTIONS

S.L.M.‐B. designed and performed the experiments, analysed data, and wrote the manuscript; A.M.B. synthesised CRP‐XL and edited the manuscript; T.R. performed in silico analysis and edited the manuscript; and M.T.H. designed experiments, analysed data, and wrote the manuscript.

## CONFLICT OF INTEREST

The authors declare no conflicts of interests.

## DECLARATION OF TRANSPARENCY AND SCIENTIFIC RIGOUR

This Declaration acknowledges that this paper adheres to the principles for transparent reporting and scientific rigour of preclinical research as stated in the *BJP* guidelines for Design and Analysis, and as recommended by funding agencies, publishers, and other organisations engaged with supporting research.

## Supporting information


**Figure S1:** **R5421 and thiodicarb do not cause GPVI shedding.** Washed platelets were treated with R5421, thiodicarb, A23187 (as positive control) or DMSO (as vehicle control) for 60 minutes. Surface GPVI expression was detected using a FITC‐conjugated antibody and analysed by flow cytometry. The median fluorescence intensity (MFI) is shown. * *p* < 0.05. No significant difference was seen between DMSO and R5421 or thiodicarb.
**Figure S2: Thiodicarb disrupts platelet Ca** ^2+^ **homeostasis.** Cal‐520‐loaded platelets were treated with R5421, thiodicarb or DMSO (as vehicle control) and fluorescence monitored for 60 minutes. The relative increase in fluorescence (F/F0) at 10 minutes (A) and 60 minutes (B) after drug treatment is shown. * *p* < 0.05 (*n* = 5).

**Table S1** Supporting Information
