# Supplementary material for: Ethaninidothioic acid (R5421) is not a selective inhibitor of platelet phospholipid scramblase activity
Source: Br J Pharmacol. 2020 Jun 30;177(17):4007–20. doi: 10.1111/bph.15152 (PMC7429475; doi:10.1111/bph.15152)
Supplement: Supplementary file 1 — Figure S1: R5421 and thiodicarb do not cause GPVI shedding. Washed platelets were treated with R5421, thiodicarb, A23187 (as positive control) or DMSO (as vehicle control) for 60 minutes. Surface GPVI expression was detected using a FITC‐conjugated antibody and analysed by flow cytometry. The median fluorescence intensity (MFI) is shown. * p < 0.05. No significant difference was seen between DMSO and R5421 or thiodicarb. Figure S2: Thiodicarb disrupts platelet Ca 2+ homeostasis. Cal‐520‐loaded platelets were treated with R5421, thiodicarb or DMSO (as vehicle control) and fluorescence monitored for 60 minutes. The relative increase in fluorescence (F/F0) at 10 minutes (A) and 60 minutes (B) after drug treatment is shown. * p < 0.05 (n = 5). [file BPH-177-4007-s001.pdf]

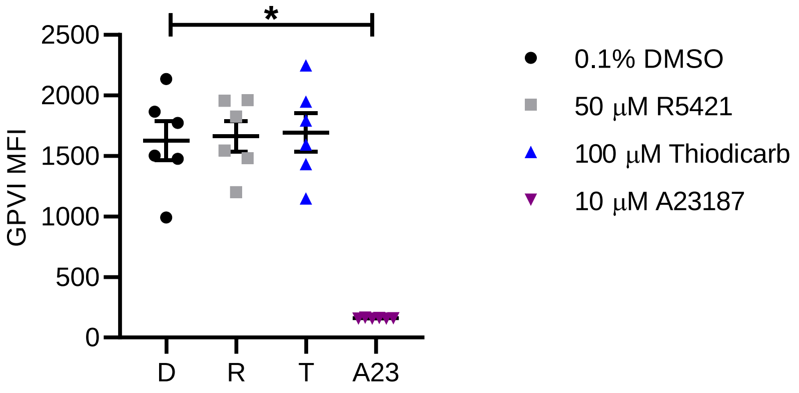

Supplementary Figure 1: R5421 and thiodicarb do not cause GPVI shedding. Washed platelets were treated with R5421, thiodicarb, A23187 (as positive control) or DMSO (as vehicle control) for 60 minutes. Surface GPVI expression was detected using a FITC-conjugated antibody and analysed by flow cytometry. The median fluorescence intensity (MFI) is shown. * $p < 0.05$. No significant difference was seen between DMSO and R5421 or thiodicarb.

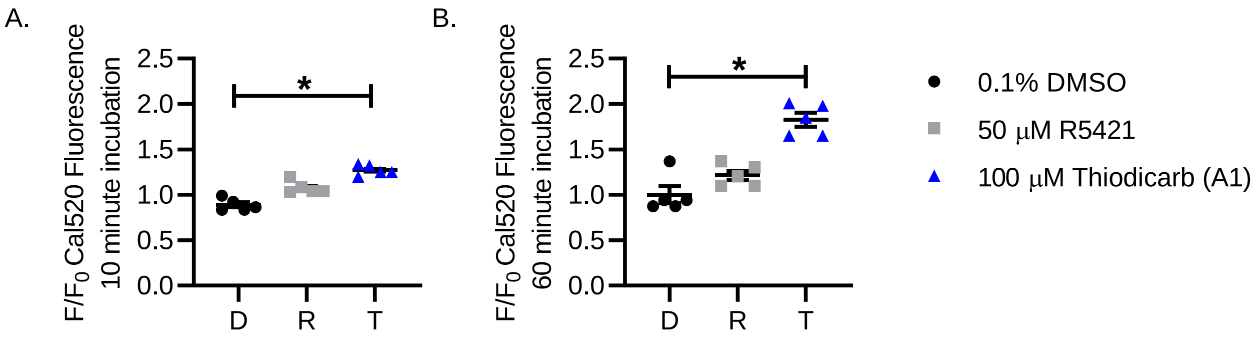

Supplementary Figure 2: Thiodicarb disrupts platelet Ca^{2+} homeostasis. Cal-520-loaded platelets were treated with R5421, thiodicarb, or DMSO (as vehicle control) and fluorescence monitored for 60 minutes. The relative increase in fluorescence (F/F0) at 10 minutes (A) and 60 minutes (B) after drug treatment is shown. * $p < 0.05$ (n = 5).
