# Supplementary material for: Ethaninidothioic acid (R5421) is not a selective inhibitor of platelet phospholipid scramblase activity
Source: Br J Pharmacol. 2020 Jun 30;177(17):4007–20. doi: 10.1111/bph.15152 (PMC7429475; doi:10.1111/bph.15152)
Supplement: Supplementary file 2 — Table S1 Supporting Information [file BPH-177-4007-s002.pdf]

| Compound | Molport ID | IUPAC | MW | Structure |
|---|---|---|---|---|
| **R5421** | | Methyl N-[butylsulfanyl(methyl)carbamoyl]oxyethanimidothioate | 250.4 | 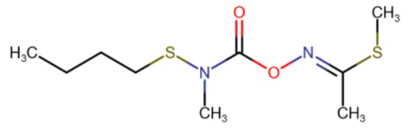 |
| **A1 Thiodicarb** | 006-131-791 | [1-(methylsulfanyl)ethylidene]amino N-methyl-N-[(3-methyl-6-oxo-5-oxa-2-thia-4,7-diazaoct-3-en-7-yl)sulfanyl]carbamate | 354.5 | 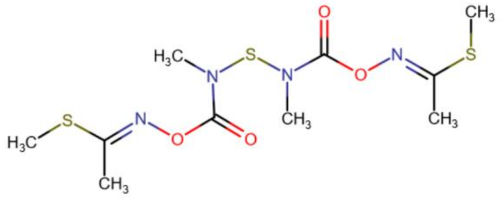 |
| **A2** | 003-035-355 | 1-(2,4-dichlorobenzoyl)-2-(ethylsulfanyl)-4,5-dihydro-1H-imidazole | 303.2 | 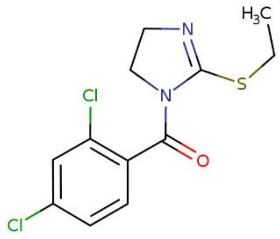 |
| **A3** | 019-921-409 | N-(but-3-yn-1-yl)cyclopropanesulfonamide | 173.2 | 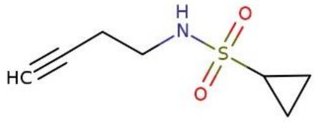 |

| | | | | |
|---|---|---|---|---|
| **A4** | 019-921-413 | N-(but-3-yn-1-yl)-1-(3-methylphenyl)methanesulfonamide | 237.3 | 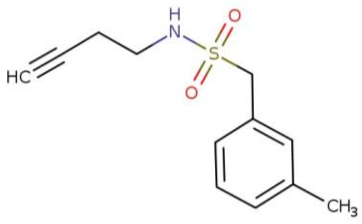 |
| **A5** | 028-614-144 | 3-methyl-N-[3-(propan-2-yloxy)propyl]-1,2,4-thiadiazole-5-carboxamide | 243.3 | 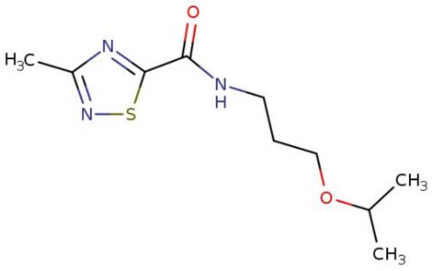 |
| **A6** | 035-671-151 | N-[2-(furan-2-yl)-2-methoxyethyl]butanamide | 211.3 | 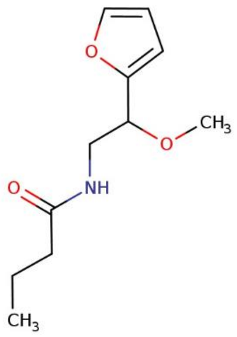 |

| | | | | |
|---|---|---|---|---|
| **A7** | 039-214-408 | 2-[(tert-butylsulfanyl)methyl]-1-cyclopropanecarbonylpiperidine | 255.4 | 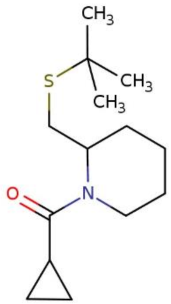 |
| **A8** | 039-214-429 | 1-{2-[(tert-butylsulfanyl)methyl]piperidin-1-yl}-2-methoxyethan-1-one | 259.4 | 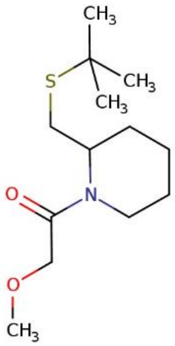 |
| **A9** | 002-892-383 | ethyl 2-(methylsulfanyl)-1,4,5,6-tetrahydropyrimidine-1-carboxylate | 202.3 | 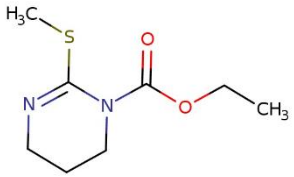 |
| **A10** | 002-905-880 | 4,4-dimethyl-1,1-bis(methylsulfanyl)pent-1-en-3-one | 204.4 | 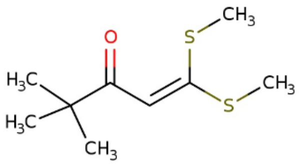 |

| | | | | |
|---|---|---|---|---|
| **A11** | 002-911-246 | 2-(ethylsulfanyl)-5,6-dimethyl-3,4-dihydropyrimidin-4-one | 184.3 | 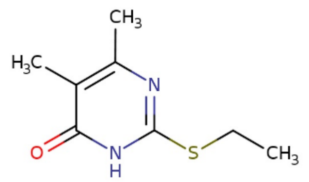 |
| **A12** | 002-913-559 | ethyl 2-{[(methylsulfanyl)methanethioyl]amino}acetate | 193.3 | 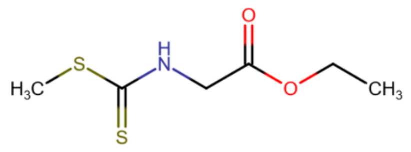 |
| **A13** | 002-466-999 | N-[4-(chloromethyl)-1,3-thiazol-2-yl]-N-ethylacetamide | 218.7 | 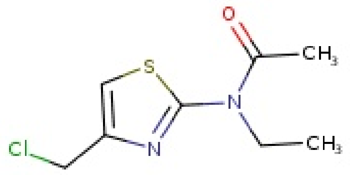 |
| **A14** | 002-470-805 | N-ethyl-N-(4-formyl-1,3-thiazol-2-yl)acetamide | 198.2 | 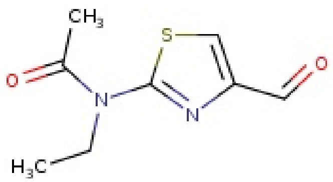 |
| **A15** | 002-479-974 | N,N-dibutylsulfamoyl chloride | 227.8 | 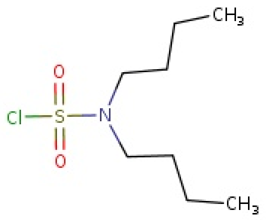 |

| | | | | |
|---|---|---|---|---|
| **A16** | 028-299-557 | N-[1-(ethylsulfanyl)propan-2-yl]-N-methyl-2-(5-methyl-1,2-oxazol-3-yl)acetamide | 256.4 | 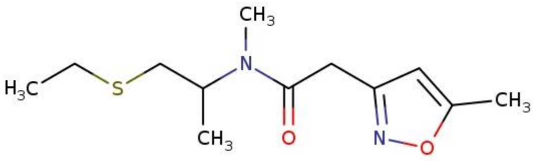 |
